# Effects of using essential oil of *Lavandula stoechas* in quail feed on growth performance, carcass characteristics, meat quality, and health status

**DOI:** 10.14202/vetworld.2020.789-795

**Published:** 2020-04-26

**Authors:** Ouafa Laghouati, Fodil Arbouche, Yasmine Arbouche

**Affiliations:** 1 Department of Veterinary, Faculty of Life and Earth Sciences, University of Chadli bendjedid, El Tarf, 36000 Algeria; 2 Department of Agronomy, Faculty of Life and Earth Sciences, University of Ghardaia, Ghardaia, 47000 Algeria; 3 Department of Agronomy, Faculty of Life Sciences, University of Sétif 1, El Bez, Sétif 19000, Algeria

**Keywords:** fat content, Japanese quail, lavender oil, microbial load, water content, zootechnical performance

## Abstract

**Aim:**

The aim of this study was to determine the effects of essential oil of *Lavandula stoechas* (Lavender) on growth performance, carcass characteristics, meat quality, and health status of quails.

**Materials and Methods:**

A group of 600 1-day-old Japanese quail chicks (*Coturnix japonica*), with an average weight of 6.8±0.2 g and a 1:1 sex ratio, were randomly assigned to four groups (150 chicks/group): three experimental groups which depend on the incorporation of lavender oil (LO) in the different phases of breeding and one control group. The experimental groups received a supplement of 1 g LO per kg of feed distributed sequentially throughout the entire 42-day breeding period.

**Results:**

No deaths were recorded throughout the breeding period. Highly significant differences were observed among the groups with regard to body weight measured on day 20 and day 42 (p<0.001 and p<0.01, respectively). The addition of LO was accompanied by reductions in liver weights; furthermore, LO had a significant effect on the pH, water content, and fat content of the meat (p<*0.05*). Administration of LO had a measurable effect on the endogenous intestinal population of *Lactobacillus*, and the bacterial load (including those of *Escherichia coli* and *Staphylococcus aureus*) was significantly reduced.

**Conclusions:**

Our results demonstrate that supplementing quail feed with LO has a profound effect on their growth as well as antimicrobial effects.

## Introduction

Antibiotics have long been used to stimulate growth of commercial poultry and to improve the production of white meat [1]. Antibiotic resistance and the harmful effect of their widespread use on both human and animal health have forced nutritionists and livestock production managers to find suitable alternatives [2]. Several recent studies have suggested that plant extracts, including essential oils, are effective replacements for antibiotics and boost zootechnical performance [3-8].

Among the aromatic plants indigenous to Algeria, different types of lavender (*Lavandula* spp.) belonging to the Labiatae (*Lamiaceae*) family have long been used in dried and natural forms for both therapeutic (antibacterial, antifungal, and antidepressant) and cosmetic purposes. It is not yet clear precisely how essential oils promote these results; however, efforts have been made toward characterizing their constituent phenolic compounds [9]. Bozkurt *et al*. [10] have suggested that the antioxidant and antimicrobial properties of the phenolic compounds found in *Lavandula stoechas* (lavender oil [LO]) have a direct effect on growth performance, carcass characteristics, and meat quality in studies focused on poultry farming. Schweitzer [11] have suggested that the breeding conditions and physiological behavior of the Japanese quail *Coturnix japonica* are similar to those of the more familiar *Gallus* spp. Some studies on quail farming have already been conducted at a national level [12,13].

In line with these works, the aim of this study was to determine the physiologic effects of LO provided to quails as a dietary supplement.

## Materials and Methods

### Ethical approval

The present study was conducted after obtaining the approval n° 05/UG/18 of the Institutional Animal Ethics Committee, Laboratory of Agriculture Department, University of Ghardaia, Algeria.

### Study period and study location

#### Animals, diets, and experimental protocol

In total, 600 1-day-old quail chicks (*C. japonica*), with an average weight of 6.8±0.2 g and a 1:1 sex ratio, were randomly assigned to four groups (150 chicks/group): One control group and three distinct experimental groups (Table-1). Each group of 150 quails was divided into five subgroups of 30 quails. All were housed on a litter of sieved wood chips in a closed building with a static ventilation system in the municipality of Tébessa, Tébessa Province, Algeria. During the 1^st^ week of the experiment, the average temperature was maintained at 39°C; the temperature was then reduced to 36°C during the growth phase and maintained at 24°C during the finishing stage. Lighting was provided 24 h/day during the first 2 weeks of the study and 12 hours for the rest of the experiment. The diets were formulated in accordance with the directives of the nutrient requirements of domestic chickens [14]. The ingredients and nutritional value of the two diets provided during each phase of breeding are indicated in Table-2. *L. stoechas* was harvested at the flowering stage from the municipality of Bougous, El Taref, Algeria. The essential oil was obtained by low-pressure steam distillation following decantation into a Florentine vase. The main constituents of the extract are included in Table-3. In the three experimental groups, LO was added to soya bean oil at a rate of 1 g/kg of feed and distributed either sequentially or not, according to the protocol in Table-1. During every phase of breeding, water and feed were distributed *ad libitum*, and any feed not eaten was weighed on a daily basis. The 150 quails in the control group were vaccinated against Newcastle disease and infectious bronchitis when the chicks were 7 days old. The quails were weighed individually on the day they were received and at the end of each phase of the breeding process. Mortality rate, food consumption, daily weight gain (DWG), consumption index (CI), and average daily intake (ADI) were determined.

**Table-1:** Constitution of the different batches with and without LO.

Breeding phase	Start-growth	Finishing
Control group	Without LO	Without LO
Experimental groups		
D_LO_	With LO	Without LO
F_LO_	Without LO	With LO
DF_LO_	With LO	With LO

LO=Lavender oil

**Table-2:** Formulae (kg/100 kg of feed) of feed distributed to the quail at start-growth (1-20 days) and during the finishing stage (21-42 days).

Composition	Start-growth	Finishing
Corn	56	62
Soya bean oil 44%	1.30	0.35
Soya meal	38.50	33.10
Dicalcium phosphate	3.30	3.60
MVS flesh	0.90	0.95
Calculated nutritional values		
Metabolisable energy (kcal/kg)	2,840	2,920
Fats (%)	4.50	3.70
Raw proteins (%)	26	24.50
Lysine (%)	1.7	1.36
Methionine (%)	0.45	0.36
Cysteine (%)	0.4	0.45

CMV: Mineral and vitamin supplement composed of, calcium: 150,700 mg/kg, sodium chloride: 332,000 mg/kg, Vitamin A: 800,000 UI, Vitamin D3: 150,000 UI, Vitamin E: 1500 mg/kg, Vitamin K: 200 mg/kg, Vitamin B1: 100 mg/kg, Vitamin B2: 450 mg/kg, Vitamin B3: 780 mg/kg, Vitamin B6: 150 mg, Vitamin B12: 1 mg/kg, PP: 1000 mg/kg, folic acid: 50 mg/kg, biotin: 1.5 mg/kg, choline chloride: 35,000 mg/kg, iron: 3600mg/kg, copper: 2250 mg/kg, zinc: 7500 mg/kg. The diets were formulated in accordance with the directives of the NRC (1994)

**Table-3:** Main components of *L. stoechas* essential oil.

Compounds	Proportion (%)
a-pinéne	0.8
Camphéne	1.5
Delta-3-caréne	0.7
1,8-cinéole	19.3
Fenchone	30.2
Camphre	20.6
Borneol	0.4
Acétate de Bornyl	3.9

*L. stoechas*=*Lavandula stoechas*

### Carcass characteristics and physicochemical analyses of the meat

At the end of the finishing phase (day 43), the body weights of 50 of the quails in each of the four groups were measured. After slaughter, the warm carcasses, cold carcasses, viscera, edible offal (liver and gizzard), feet, heads, and feathers were all weighed. The meat removed from each quail was ground and homogenized. The moisture, ashes, protein content, and fat content were determined and calculated in accordance with AOAC [15]. The pH was measured as previously described by Korkeala *et al*. [16] in 2 g of muscle homogenate (*Pectoralis superficialis*) in 18 ml of buffered iodoacetate. The pH was also measured at 1, 24, and 72 h postmortem by inserting the electrode of a pH meter directly into the pectoral muscle (~2 cm deep), as previously described by El Rammouz [17].

### Bacteriological analysis

The droppings from each group were removed at the end of each breeding cycle. Microbiological analysis was conducted as per the guideline of AFNOR [18] to detect *Salmonella*. Furthermore, the antimicrobial activity of LO was evaluated by counting the colony-forming units, as previously described by Khaksar [19].

### Statistical analysis

The different results were processed using the Microsoft Excel spreadsheet. The statistical analysis and comparison of averages between the different dietary schemes were conducted by means of the one-way analysis of variance (ANOVA) test using the Statistical Package for the Social Sciences software (SPSS Inc. version 25 Chicago, USA), then completed by means of the Student-Newman-Keuls and Duncan test if the ANOVA test displayed a significant difference from the error risk of 5% (p<0.05).

## Results

### Zootechnical performance

No mortality was reported among any of the groups during the entire 42-day breeding period. Introduction of the LO supplement led to a significant reduction in the final live weights and the DWG of the D_LO_ group compared with that of the control group; by contrast, we observed significant increases in both final live weights and DWG of the F_LO_ and DF_LO_ groups compared with those of the controls (p<0.002; Table-4). The DWG over days 1-42 was relatively high in the DF_LO_ group, intermediate in the F_LO_ and control groups, and minimal in the D_LO_ group (p<0.005). The control and DF_LO_ groups had similar DWG over days 21-42, whereas these measurements in the F_LO_ and D_LO_ groups were significantly higher, particularly in the D_LO_ group (p<0.002).

**Table-4:** Effect of introducing LO supplements on the weight growth and average DWG in quails.

Groups	Control	D_LO_	F_LO_	DF_LO_	SEM	p-value
Start-growth phase						
Initial weight (g)	7	6.5	7	7	0.254	0.129
Body weight at 20 days	111^a^	97^b^	106^ab^	113^a^	10.83	0.019
DWG 1-20 days (g/d/subject)	5.21^b^	4.54^d^	4.95^c^	5.33^a^	0.951	0.006
Finishing phase						
Body weight at 42 days	205^b^	201^c^	207^ab^	209^a^	15.15	0.002
DWG 21-42 days (g/d/subject)	4.48^c^	4.95^a^	4.81^b^	4.57^c^	1.18	0.002
DWG 1-42 days (g/d/subject)	4.84^b^	4.74^c^	4.87^b^	4.93^a^	1.58	0.005

LO=Lavender oil, DWG=Daily weight gain. In each line, the numbers followed by the same exponents do not differ significantly at p<0.05

The ADI of the DF_LO_ group was the highest among all the four groups at 0.57 kg/quail (p<0.03) during the initial growth phase; the ADI during the finishing phase (1.02 kg/quail) was equivalent to that of the D_LO_ group (Table-5). During the entire breeding phase, the ADI of the DF_LO_ group was highest (1.59 kg/quail; p<0.001); and the CI was highest for the control group (5.74) and lowest for the D_LO_ group (4.46; p<0.01). The F_LO_ and DF_LO_ groups displayed intermediate consumption indices.

**Table-5:** Effect of introducing LO supplements on the ADI and the CI in quails.

Groups	Control	D_LO_	F_LO_	DF_LO_	SEM	p-value
ADI (kg/subject)						
1-20 days	0.40^c^	0.38^c^	0.52^b^	0.57^a^	0.125	0.03
21-42 days	1.15^a^	1.02^b^	0.97^c^	1.02^b^	0.95	0.02
1-42 days	1.55^b^	1.0^d^	1.49^c^	1.59^a^	0.23	0.001
Consumption index						
1-20 days	4.44^c^	4.49^c^	4.96^b^	5.05^a^	1.78	0.03
21-42 days	6.38^a^	4.93^b^	4.69^c^	4.89^b^	1.65	0.02
1-42 days	5.74^a^	4.46^d^	4.78^c^	4.94^b^	1.08	0.001

LO=Lavender oil, ADI=Average daily intake, In each line, the numbers followed by the same exponents do not differ significantly at p<0.05

### Carcass characteristics and meat quality

The carcass characteristics and physicochemical properties of the meat varied significantly in response to the addition of LO to the diet (p<0.05; Table-6). The weights of the gizzard, legs and head; pH measurements at 1 h postmortem; and protein levels were not affected by the introduction of LO. However, LO in the feed was associated with increases in both warm and cold carcass weights, carcass yield, and feather weights, as well as in the water content of the meat in the DF_LO_ group (DF_LO_ > control; p<0.05). The weight of the livers, the pH at 24 and 72 h postmortem, and the ash content of the meat were also modified significantly in response to the addition of LO (p<0.05 vs. control group). Furthermore, a considerable reduction in the weight of the viscera was observed among quails in the F_LO_ group compared with those in the other groups. The fat content of the meat increased significantly with the addition of LO to the diet in all three experimental groups, although these results did not reach statistical significance.

**Table-6:** Effect of introducing LO supplement on the carcass characteristics and physicochemical properties of meat in quails.

Groups	Control	D_LO_	F_LO_	DF_LO_	SEM	p-value
Carcass characteristic						
Live weight (g)	207	205	207	208	1.24	NS
Warm carcass weight (g)	155^b^	154^b^	154^b^	159^a^	1.56	0.04
Warm carcass yield	0.75^b^	0.75^b^	0.74^b^	0.76^a^	1.66	0.01
Cold carcass weight (g)	154^b^	152^b^	153^b^	158^a^	1.54	0.03
Cold carcass yield	0.74^b^	0.74^b^	0.74^b^	0.76^a^	1.89	0.02
Weight of viscera (g)	30^a^	29^a^	27^b^	30^a^	0.63	0.02
Viscera yield	0.14^a^	0.14^a^	0.13^b^	0.14^a^	0.03	0.01
Weight liver (g)	6.60^a^	5.50^b^	5.30^b^	5.40^b^	1.32	0.03
Liver yield	0.032^a^	0.027^b^	0.026^b^	0.026^b^	0.003	0.01
Weight gizzard (g)	5.20	5.16	5.18	5.15	0.21	NS
Gizzard yield	0.025	0.025	0.025	0.025	0.002	NS
Weight of head (g)	12.60	12.40	12.50	12.30	1.02	NS
Head yield	0.061	0.060	0.060	0.059	0.001	NS
Weight of feet (g)	4.2^a^	4.00	4.00	4.10	1.25	NS
Feet yield	0.020	0.019	0.019	0.020	0.02	NS
Weight of feathers (g)	14.40^c^	15.00^b^	15.10^b^	15.40^a^	2.01	0.02
Feathers yield	0.070^c^	0.073^b^	0.073^b^	0.074^a^	0.05	0.02
Physical-chemical properties of the meat						
pH 1 h postmortem	6.41	6.44	6.42	6.40	1.23	NS
pH 24 h postmortem	7.42^a^	7.37^b^	7.31^b^	7.33^b^	1.54	0.04
pH 72 h postmortem	8.20^a^	7.97^b^	7.96^b^	7.98^b^	0.98	0.03
Water content (% of DM)	52.73^b^	46.85^c^	46.98^c^	55.90^a^	2.32	0.01
Ash (% of DM)	1.36^a^	1.29^b^	1.21^b^	1.07^c^	0.25	0.02
Protein content (% of DM)	19.20	19.17	19.18	19.21	2.65	NS
Fat level (% of DM)	5.17^b^	5.34^a^	5.31^a^	5.32^a^	1.36	0.04

LO=Lavender oil, In each line, the numbers followed by the same exponents do not differ significantly at p<0.05

### Antimicrobial activity of LO

The evaluation of antimicrobial activity of LO revealed a significant difference among the groups during the breeding phases (Table-7). A significant reduction in *Lactobacillus* spp. was observed throughout; reductions in *Staphylococcus aureus* and *Escherichia coli* were observed in the DF_LO_ group compared with the other experimental groups and the control group (p<0.05). The sequential addition of LO provided no conclusive results with regard to the microbial load.

**Table-7:** Effect of introducing LO supplement on the development of seeds in quail droppings. Figures presented in log UFC/g.

Groups	Control	D_LO_	F_LO_	DF_LO_	SEM	p-value
Start of growth phase						
*E. coli*	9.50^a^	9.35^a^	9.51^a^	8.34^b^	0.254	0.012
Lactobacilli	7.10^a^	7.10^a^	7.13^a^	6.11^b^	1.83	0.019
Salmonellae	Absence	Absence	Absence	Absence	/	/
*S. aureus*	7.30^a^	7.35^a^	7.13^b^	7.10^b^	2.08	0.01
Finishing phase						
*E. coli*	9.79^a^	9.66^a^	9.73^a^	7.60^b^	12.54	0.008
Lactobacilli	7.34^a^	7.33^a^	7.11^b^	7.12^b^	1.18	0.002
Salmonellae	Absence	Absence	Absence	Absence	/	/
*S. aureus*	7.35^a^	7.30^a^	7.10^b^	7.09^b^	1.98	0.01

*E. coli*=*Escherichia coli*, *S. aureus*=*Staphylococcus aureus*, LO=Lavender oil, In each line, the numbers followed by the same exponents do not differ significantly at p<0.05

## Discussion

The addition of LO to quail feed throughout the breeding period (days 1-42) and specifically during the finishing period (days 21-42) resulted in improvements in both final live weight and DWG. This trend is similar to that reported by Nasiri-Moghaddam *et al*. [20], who reported that the addition of 350 ppm of essential oil of lavender (*Lavandula angustifolia*) to the diet of broiler chickens resulted in increased live weights during the 22-42 day interval. By contrast, Küçükyilmaz *et al*. [9] reported that diets supplemented with 24 or 48 mg of EOLS per kg feed had no effect on the final body weight of 39-day-old broiler chickens. In accordance with these findings, the study conducted by Salajegheh *et al*. [21] demonstrated that adding 1% herbal powder of *L. angustifolia* to broiler feed resulted in significant increases in weight gain during all breeding phases (growth and finishing). In their recent study, Adaszyńska-Skwirzyńska and Szczerbińska [22] also showed that addition of essential oil of lavender (*L. angustifolia*) to the drinking water of broiler chickens had a positive effect on final live weight and DWG during the entire breeding period (days 1-42), most notably on days 22 and 24.

The addition of LO during the entire 42-day breeding period resulted in a reduction in both the amount eaten and the CI. Interestingly, Mokhtari *et al*. [23] and Küçükyilmaz *et al*. [9] found that addition of LO had no effect on either of these two parameters, although Bozkurt *et al*. [10] found that addition of essential oil derived from plants of the family Labiatae stimulated the growth in broiler chickens. Furthermore, the quantity of LO distributed (1 g/kg feed) may have an effect on these results [24]. Kanyinji and Moonga [25] reported that high consumption indices could be linked to wasteful behaviors. Finally, while there are only a few studies that address this point, the addition of LO to quail feed generates contradictory results with regard to other essential oils [19,26,27].

We also found that the addition of LO during all breeding phases resulted in significant differences in the characteristics of the carcass and the physicpchemical properties of the meat. These findings included a significant increase in both warm and cold carcass weights, carcass yield, and feather weights; interestingly, we observed no effect on live weight or the weights of the feet, head and gizzards, similar to results reported previously [9]. The effect of LO on the weight and development of the gizzard may be neutral due to the nature of its physiological function [27,28], although several groups have noted that administration of LO resulted in smaller livers. It is important to recognize that these findings are not specific to LO [29], although Biricik *et al*. [26] reported that administration of essential oil of myrtle (EOM) had no significant effect on liver weight.

Adding LO to the quails’ diet had no effect on meat pH determined at 1 h, but resulted in a significant reduction in pH at both 24 and 72 h postmortem. These results stand in contrast to those of Biricik *et al*. [26] who reported that addition of EOM had no significant effect on the pH of quail meat. By contrast, Aksu *et al*. [30] observed a significant increase in the pH of breast filets in response to EOM and concluded that the final pH was affected by numerous factors including age, sex, breeding methods, feed additives, stress before slaughter, hormonal status, muscle morphology, and glycogen content.

We also found that administration of LO throughout the breeding process resulted in an increase in fat content and water content accompanied by a decrease in ash content of the quail meat; interestingly, we observed no effect on protein content. This result was similar to that reported by Bolukbasi *et al*. [31] but was contradictory to that of Fotea *et al*. [32] who reported that this intervention resulted in no changes whatsoever in the chemical composition of broiler chicken meat. Alleman *et al*. [33] also noted several varied effects of essential oils on the physicochemical properties of meat.

Finally, we examined the effect of LO on gut bacterial content. We noted the complete absence of contaminating *Salmonella* spp.; bacteria of the genera *Lactobacillus* are components of the endogenous bacterial microbiome. Ramdane and Guitarni [34] reported that the quail’s digestive tract is sterile before birth and that the appearance of intestinal flora depends on the nature of the immediate environment of the egg at the time of hatching. This trend has also been observed by Fuller [35], who detected lactobacilli in the digestive tract of chicks beginning on day 3 after hatching. Furthermore, the number of colony-forming units of *E. coli* and *S. aureus* was diminished in the experimental groups. These findings are consistent with those of Boughendjioua [36], who observed that *E. coli* are sensitive to LO. Several other studies, notably those of Derwich *et al.*[37] and Bari *et al*. [38], reported a high level of resistance to LO among Gram-negative bacteria as opposed to Gram-positive bacteria. These results might be explained by the relative volatility of essential oils and likewise, due to the lipopolysaccharide content at the Gram-negative bacterial surface that might serve as an effective barrier to incoming biomolecules [35]. This trend was reported by Dorman and Deans [39], who noted that the different components of essential oils demonstrate varying degrees of activity against Gram-negative versus Gram-positive bacteria; the chemical composition of essential oils may vary according to both intrinsic and extrinsic factors [40]. According to Botsoglou *et al*. [41], healthy, well-fed chicks do not typically respond to supplements that promote growth when they are housed with only basic hygienic management.

## Conclusion

Supplementation of quail feed with LO facilitated specific improvements in zootechnical performance, carcass weight, and carcass yield, as well as the physicochemical properties of the quail meat. Microbial loads were reduced and the health status of the quails was improved.

## Authors’ Contributions

OL prepared the ground conditions and collected the data. YA performed the analysis of the data. FA designed the study and drafted the manuscript. All authors have read and approved the final manuscript.
